# Binge eating symptomatology, BMI, and health

**DOI:** 10.1186/2050-2974-3-S1-O59

**Published:** 2015-11-23

**Authors:** Jennifer Mackenzie, Lynne Harris

**Affiliations:** 1 Australian College of Applied Psychology, Australia

## Research Aims/Questions

Binge Eating Disorder (BED) has been recognised as a diagnostic classification in DSM-5. There is limited research into subtypes of BED. Although BED does not have a weight criteria many people with BED are overweight or obese (Mason & Lewis, 2014). This study compared people with elevated binge eating symptomatology who varied in BMI on measures of physical and psychological health.

## Methodology

From a sample of 427 people who completed an online questionnaire, 211 with scores in the bingeing range on the Binge Eating Scale (BES) were selected. Of these, 110 (n=106 female) had BMI in the normal range and 101 (n=94 female) had BMI in the overweight/obese range. No significant differences were found between BMI groups on measures of binge eating, negative affect, satisfaction with life or history of psychological treatment. Those in the normal BMI range were more likely to report that their health was good or excellent (Χ2 (4, N=211)=12.43, p=.014).

## Conclusion

Much previous research with BED has examined overweight and obese treatment seeking samples, limiting the generalisability of findings to those in the normal BMI range. The present findings will be discussed in terms of their implications for treatment of individuals with BE symptomatology.

